# Hotspots and frontiers in pulmonary arterial hypertension research: a bibliometric and visualization analysis from 2011 to 2020

**DOI:** 10.1080/21655979.2022.2100064

**Published:** 2022-07-26

**Authors:** Zhen He, Lei Dai, Yuyue Zuo, Yu Chen, Hongjie Wang, Hesong Zeng

**Affiliations:** a Division of Cardiology, Department of Internal Medicine, Tongji Hospital, Tongji Medical College, Huazhong University of Science and Technology, Wuhan, China; b Hubei Provincial Engineering Research Center of Vascular Interventional Therapy, Wuhan, China; c Department of Dermatology, Wuhan No. 1 Hospital, Tongji Medical College, Huazhong University of Science and Technology, Wuhan, China

**Keywords:** Pulmonary arterial hypertension, bibliometric analysis, visualization, selexipag, treprostinil

## Abstract

Pulmonary arterial hypertension (PAH) is a group of devastating and progressive disorders, resulting in relentless increases in pulmonary vascular resistance. The number of studies related to PAH has been increasing in recent years. Our study aims to illustrate trends in PAH research over the past decade using bibliometric analysis. Science Citation Index-Expanded was adopted to search studies concerning PAH between 2011 and 2020. The bibliographic information was converted and analyzed automatically using a bibliometric package in R software and citespace. The annual quantity of publications on PAH showed an overall increase last decade. The United States was the most prolific country with 2,479 publications, and it was also the country that cooperated most with other countries. Hôpital Bicêtre made important research achievements on PAH and was a leader in study cooperation. Marc Humbert led the PAH field by publishing 150 articles in the past decade. During the past decade, there was a close transnational relation among countries or regions, institutions and authors. Further, *Circulation* was the most cited journal, followed by the *Journal of the American College of Cardiology* and the *American Journal of Respiratory and Critical Care Medicine*, with 3,895, 3,406, and 3,170 citations, respectively. The global research status and trend of PAH are deeply understood for the first time using bibliometric and visual methods, and the results of our study bring us a valuable reference for clinical researchers.

## Highlights

- This is the first study to illustrate trends in pulmonary arterial hypertension research using bibliometric analysis.
- Our study provides extensive and in-depth directions for researchers.
- Our study may benefit further researches on the etiology, diagnosis, and treatment of pulmonary arterial hypertension.

## Graphical abstract

**Figure:**
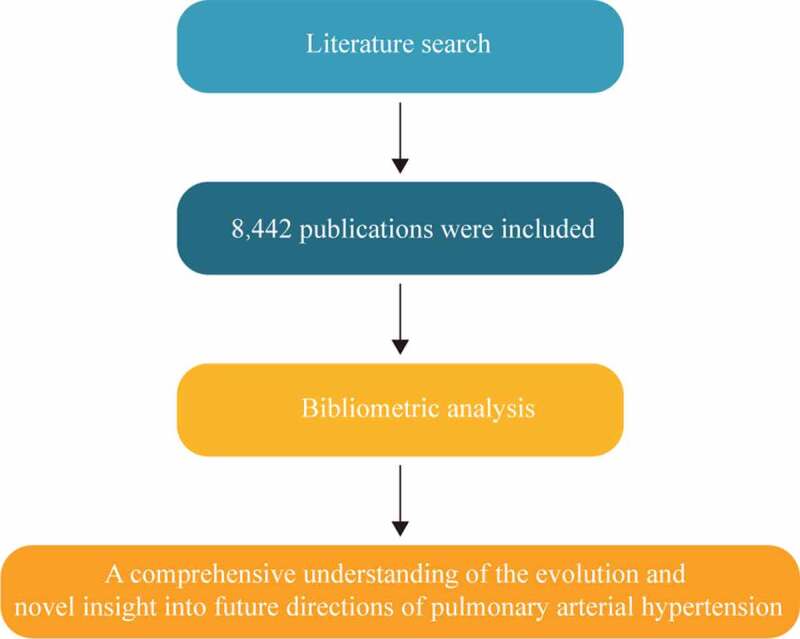

## 1. Introduction

Pulmonary hypertension (PH), characterized by mean pulmonary artery pressure greater than or equal to 25 mmHg in the resting state [1], refers to a progressive disease typified by increased pulmonary blood pressure, abnormal thickening of the pulmonary arteries, and right ventricular remodeling. It is divided into the pre-capillary form or post-capillary form haemodynamically based on left ventricular filling pressure. Pulmonary arterial hypertension (PAH), or group I PH, is a group of devastating and progressive disorders manifested by pulmonary vascular remodeling and inappropriate vasoconstriction, resulting in relentless increases in pulmonary vascular resistance, which ultimately lead to right heart failure and death [2–5]. It can be classified into four subtypes based on the current classification criteria [6,7](BOX 1). PAH is a rare disease with an approximate minimum incidence of 15 cases per million individuals [8]. Before the advent of new therapies, the estimated median survival for patients with idiopathic or familial pulmonary hypertension was 2.8 years, and their 1-year, 3-year, and 5-year survival rates were 68%, 48%, and 34%, respectively [9]. The diagnosis and treatment of PAH have improved in recent years, however, the main treatment for PAH remains symptomatic treatment, which can not fundamentally improve the survival rate of these patients. Therefore, its mortality is not optimistic [7]. It is complex to track the generation and evolution of knowledge in PAH in the past 10 years. On the one hand, the field has developed rapidly in the past decade. On the other hand, PAH is difficult to be deeply understood due to its heterogeneous disease with multiple factors. Box 1.Pulmonary arterial hypertension1 Idiopathic2 Heritable2.1 BMPR2 mutation2.2 Other mutations3 Drugs and toxins induced4 Associated with:4.1 Connective tissue disease4.2 Human immunodeficiency virus (HIV) infection4.3 Portal hypertension4.4 Congenital heart disease4.5 Schistosomiasis

Bibliometric analysis is a convenient novel method to quantitatively and qualitatively analyze publications [10]. Based on the method, researchers can quickly and deeply explore the topic evolution, main research areas, and promising research directions of a certain research area [11].

Bibliometric analysis might be an appropriate choice for providing detailed knowledge structure and development of PAH. Accordingly, our study aims to illustrate trends in PAH research over the past decade using bibliometric analysis to outline the evolution of PAH. In turn, the data obtained from the analysis provide us not only a comprehensive understanding of the evolution but also novel insight into future directions related to advances in PAH.

## 2. Materials and methods

### 2.1 Literature search and screening

**Figure 1.**
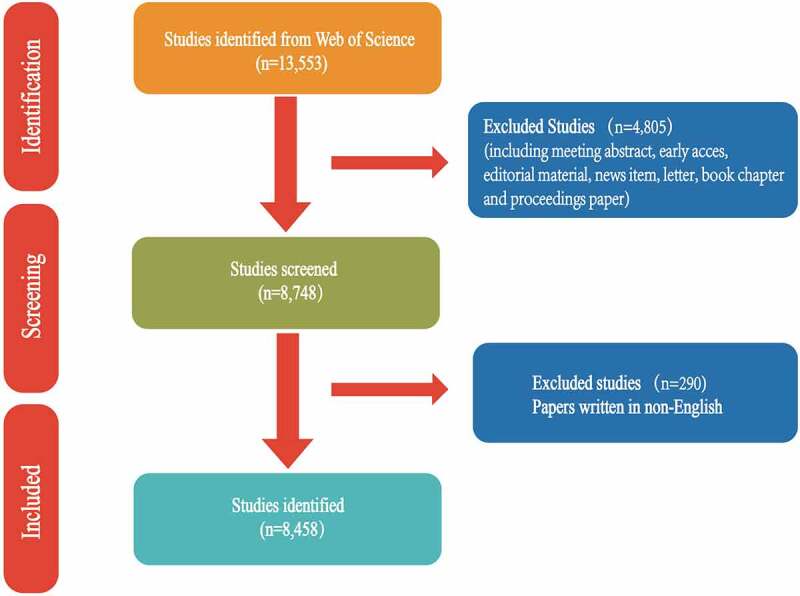
Flowchart of data filtration processing and excluding publications.

It is acknowledged that the Web of Science Core Collection (WoSCC) database is a broadly used database for performing bibliometric analysis [12–14]. We adopted the Science Citation Index-Expanded (SCI-E) to search studies concerning PAH until 3 July 2021. The search strategies were shown as follows: [TS = (‘pulmonary arterial hypertension’)] AND [Language = (English)]. TS meant ‘TOPIC’ as indicated by WoSCC. We did not impose any additional restrictions on this strategy for the relevant records. Original articles and reviews with full manuscripts that referred to PAH as the main topic were included. Two authors (ZH and LD) identified papers published from 2011 to 2020 independently. Discrepancies in the process were resolved by two authors through discussions until they came to an agreement. If necessary, a third author (YZ) was consulted to arbitrate. Figure 1 shows the detailed selection process.

### 2.2 Data analyses and visualization

After identifying all the eligible publications, the bibliographic information was converted and analyzed automatically by using the bibliometric package (Version 3.0.0) in R software (Version 3.6.1) [15]. The information extracted from each study included title, authors, keywords, institutions, countries and regions, total citation (TC), year of publication, journal, impact factor, and so on. Available information of the records, i.e. bibliographic information, were imported to CiteSpace5.7. R2, a Java-based software to present the structure and distribution of scientific knowledge [16]. In addition, collaboration network analysis of institutions and authors was visualized by Vosviewer, which was developed by Waltman *et* *al* [17]. And, the geographical distribution map of national publications was generated in Microsoft Excel 2019 (Microsoft Corp., Redmond, WA, USA).

### 2.3 Ethics

This study drew on available published articles and did not involve animal or human experiments. Thus, ethics approval from an institutional review board was not required. No authors were contacted for further information regarding their publications.

## 3. Results

With the progress of science and technology, scientific knowledge is constantly produced and replaced. In recent years, remarkable achievements have been made in the field of PAH. While enjoying all this scientific knowledge, researchers are faced with the dilemma of how to select the knowledge they are interested in from the complex information. The purpose of this study is to explore the general situation of PAH in recent years from the bibliometrics perspective so that researchers can have a comprehensive understanding of this field. We will describe the development of PAH in recent ten years from the perspectives of country, institution, author and keywords.

### 3.1 Annual quantitative distribution of publications

**Figure 2.**
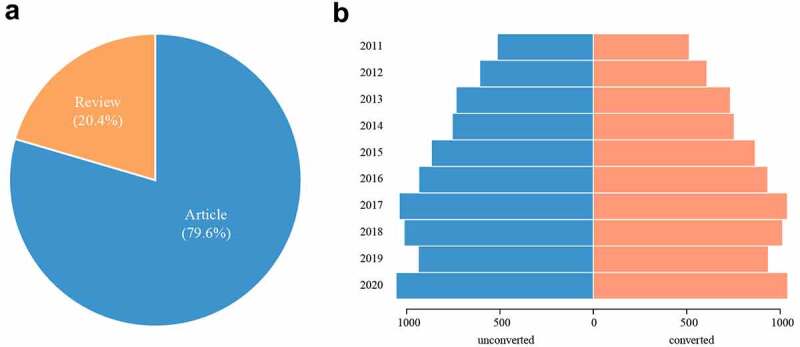
Annual quantity and the types of literature published on pulmonary arterial hypertension (PAH). (a) the types of and (b) annual numberpublished publications in PAH research from 2011 to 2020.

8,458 publications associated with PAH were identified in the WoSCC from 2011 to 2020, of which 6,720 (79.6%) were indexed as ‘article’ and 1,722 (20.4%) as ‘review’ (Figure 2(a)). The number of papers published every year is an important indicator that reflects the speed of subject knowledge and presents the trend in the field [18]. The annual quantity of literature in the PAH field is shown in Figure 2b. We can intuitively find that the number of PAH-related literature increased year by year from 2011 to 2017, followed by a slight decrease in 2018 and 2019, and then returned to peak in 2020, indicating the growing interest in PAH-related research. However, given a full-blown COVID-19 outbreak in 2020, the annual quantity of publications that year did not reflect the true picture. Thus, we corrected this amount, in which case the publications related to COVID-19 were excluded, and we can find that the growth trend of the revised version is similar to that of the initial (Not exclude COVID-19-related papers) (Figure 2(a)).

### 3.2 Citation count of the 10 most influential publications

TC is a significant indicator which refers to the number of times an article has been cited by other articles since it was published and mirrors the value of an article to some extent. According to the citation analysis of the documents, the top 10 high citations were listed in Table 1. The TC of the top 10 cited papers varied from 3,309 (‘2015 ESC/ERS Guidelines for the diagnosis and treatment of pulmonary hypertension’) to 579 (‘Oxygen Sensing, Hypoxia-Inducible Factors, and Disease Pathophysiology’). Intriguingly, eight of these 10 studies focused on clinical problems of PAH, and the rest concerned oxygen sensing and its related diseases. The earliest published study on the list, which focused on the advances in oxygen sensing, was published in the *New England Journal of Medicine* (NEJM) in 2011. It indicated that some oxygen-sensing molecules including hypoxia-inducible factor 1 α and hypoxia-inducible factor 2 α played a critical role in the development of PAH. One of the articles on the list was published in 2016, and it was still the most cited since it was a clinical guideline on PAH and provided practical information and evidence for researchers and doctors.

**Table 1.** Citation count of the 10 most influential publications.

Rank	Article	Total Citation	Publication Year	Journal
1	2015 ESC/ERS Guidelines for the diagnosis and treatment of pulmonary hypertension	3,309	2016	European Heart Journal
2	Updated Clinical Classification of Pulmonary Hypertension	3,074	2013	Journal of the American College of Cardiology
3	2013 Classification Criteria for Systemic Sclerosis An American College of Rheumatology/European League Against Rheumatism Collaborative Initiative	1,498	2013	Arthritis and Rheumatism
4	Definitions and Diagnosis of Pulmonary Hypertension	1,004	2013	Journal of the American College of Cardiology
5	2016 ESC Position Paper on cancer treatments and cardiovascular toxicity developed under the auspices of the ESC Committee for Practice Guidelines	909	2016	European Heart Journal
6	2013 classification criteria for systemic sclerosis: an American college of rheumatology/European league against rheumatism collaborative initiative	892	2013	Annals of the Rheumatic Diseases
7	Hemodynamic definitions and updated clinical classification of pulmonary hypertension	826	2019	European Respiratory Journal
8	Macitentan and Morbidity and Mortality in Pulmonary Arterial Hypertension	746	2013	New England Journal of Medicine
9	Mechanisms of Disease Oxygen Sensing, Homeostasis, and Disease	606	2011	New England Journal of Medicine
10	Oxygen Sensing, Hypoxia-Inducible Factors, and Disease Pathophysiology	579	2014	Annual Review of Pathology: Mechanisms of Disease

### 3.3 Countries or regions, institutions, and authors

**Figure 3.**
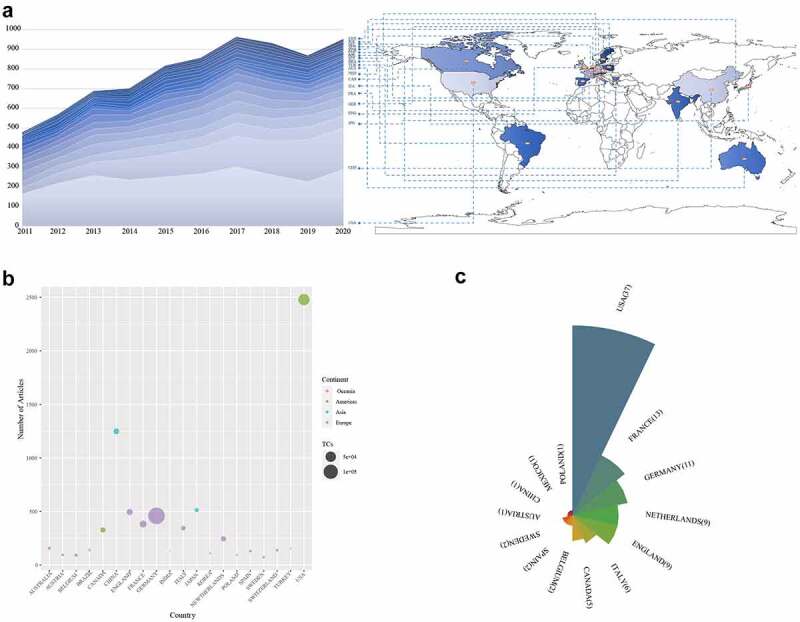
The distribution of countries or regions publishing PAH research. (a) Distribution of PAH literature in a world map, (b) bubble map of the number of publications related to PAH derived from different countries or regions from 2011 to 2020, (c) rose map of the number of publications related to PAH in analyzing the top 100 cited papers produced by different countries or regions from 2011 to 2020.

Visualized information helps us to understand distinguished teams and clarify cooperation between countries, institutions, and authors. The top 20 productive countries/regions are presented in Figure 3a. The top 20 countries/regions were located on five continents, of which 12 were located in Europe. The United States was the most prolific country in PAH research, followed by China and Japan (Figure 3(b)). Additionally, only the three countries mentioned above posted more than 500 articles in the previous decade. Only the number of publications derived from China has increased almost every year (Figure 3(a)). Intriguingly, China ranked second in the number of published articles but fifth in TCs (Figure 3(b)). In contrast, the United States ranked first in both the number of articles published and the number of TCs. In analyzing the 100 most cited publications, we observed that the United States remained at the top, but was followed by France, Germany, and the Netherlands rather than China and Japan (Figure 3(c)).

**Figure 4.**
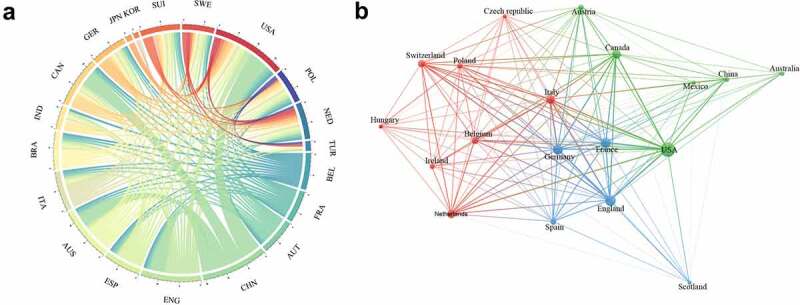
Collaboration network of countries or regions. (a) The cooperation between countries or regions that published articles related to PAH from 2011 to 2020. (b) The cooperation relationships of countries or regions that published the top 100 cited articles.

A close partnership among countries or regions was exceedingly common during the investigation period. Figure 4 depicts the partnership among countries that published PAH-related papers and/or the 100 most influential articles, suggesting the close cooperation among the various countries and regions. In consistency with the fact that the United States contributed the most articles, it was also the country that cooperated most with other countries or regions, followed by China and England. In addition, the United States collaborated particularly closely with China (Figure 4(a)). In analyzing the 100 most cited publications, however, China ranked 13th in cooperation with other countries or regions (Figure 4(b)).

**Figure 5.**
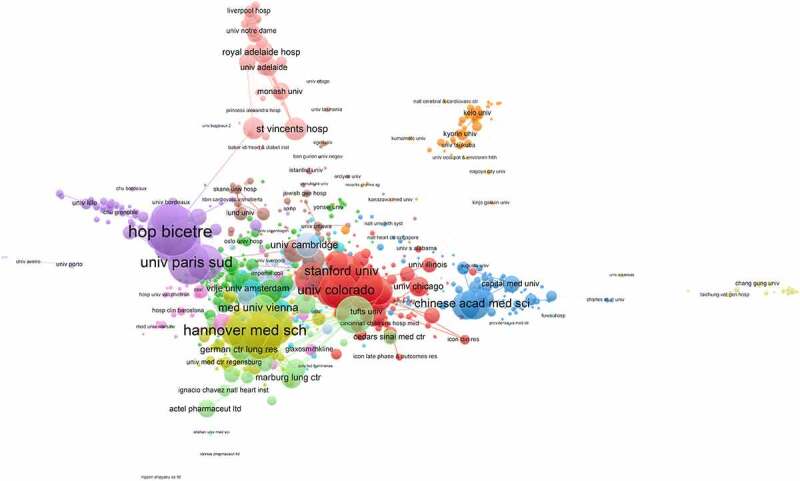
Collaboration network of institutions. The cooperation relationships of institutes that published articles related to PAHfrom 2011 to 2020. Different colors represent different clusters.

As noted in Table 2, the top 10 institutions that published the highest number of articles include Hôpital Bicêtre, Vanderbilt University, and the Chinese Academy of Medical Science with 124, 122, and 117, respectively. The top 10 prolific institutions included two French institutions, six American ranked institutions were institutions, one Chinese institution, and one German institution. Although the United States had the most institutions in the top 10, none of them ranked first. It was intriguing that the institutions ranking changed dramatically when analyzing the 100 most cited publications. The top three ranked institutions were Université Paris-Sud, Mayo Clin, and Hannover Med Sch, respectively (Table 3). Furthermore, the top 10 was composed of two French institutions, five American institutions, two German institutions, and one Italian institution. However, Chinese institutions were absent from the list. Cooperation among different institutions is considered crucial traction to promote the development of successful large-scale research. To this point, there appeared to be a close partnership among different organizations from various countries and regions. During the last decade, Hôpital Bicêtre cooperated with practically every authoritative scientific institution in PAH research except the Chinese Academy of Medical Sciences, in contrast to the Chinese Academy of Medical Sciences, which collaborated almost exclusively with influential institutions in China (Figure 5).

**Table 2.** Top 10 institutions that published the highest number of articles from 2011 to 2020.

Rank	Institution	Number of Publications	Country
1	Hôpital Bicêtre	124	France
2	Vanderbilt University	122	USA
3	Chinese Academy of Medical Sciences	117	China
4	University of Colorado	115	USA
5	Stanford University	110	USA
6	University of Pittsburgh	101	USA
7	Université Paris-Sud	97	France
8	Mayo Clinic	97	USA
9	Hannover Medical School	97	Germany
10	University of Pennsylvania	89	USA

**Table 3.** Top 10 institutions that published the highest number of articles when analyzing the 100 most cited articles from 2011 to 2020.

Rank	Institution	Number of Publications	Country
1	Université Paris-Sud	13	France
2	Mayo Clinic	13	USA
3	Hannover Medical School	11	Germany
4	University of Michigan	10	USA
5	University of Colorado	10	USA
6	Hôpital Bicêtre	9	France
7	University of Giessen	8	Germany
8	Columbia University	8	USA
9	Ctr Chirurg Marie Lannelongue	8	Italy
10	Baylor College of Medicine	8	USA

**Figure 6.**
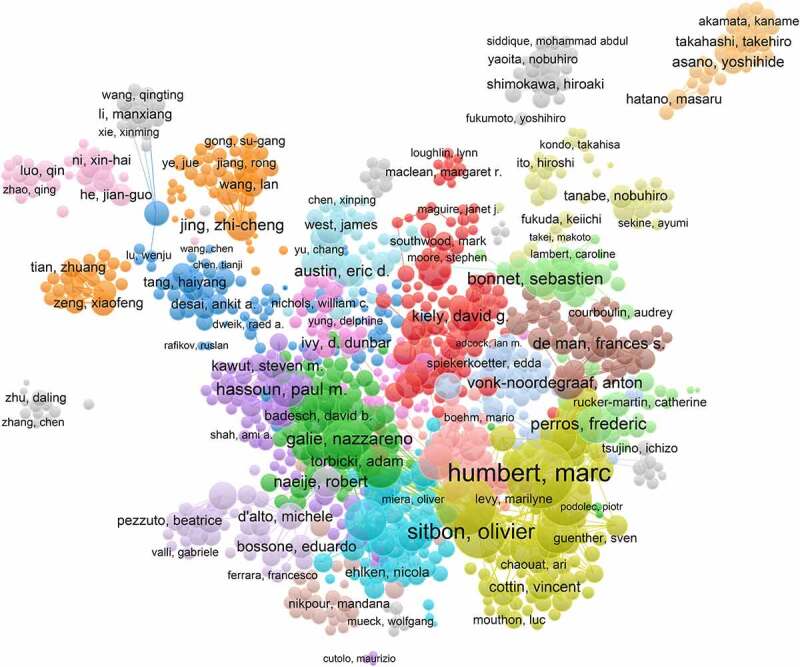
Collaboration network of authors. The cooperation relationships of authors that published articles related to PAH from2011 to 2020. Different colors represent different clusters.

To identify the most influential experts in PAH over the past 10 years, all authors were ranked according to their number of publications. The top 10 prolific authors in the area of PAH are shown in Table 4. Among them, the top 3 authors were Marc Humbert, Gerald Simonneau, and Olivier Sitbon, who published 150, 84, and 62 articles, respectively. Notably, none of the authors on this top 10 list came from the Asian region. In analyzing the 100 most influential studies on PAH research, Marc Humbert and Gerald Simonneau were still in the top three, but their rankings had been reversed, that is, Gerald Simonneau was ranked first and Marc Humbert was ranked second (Table 5). The author’s collaboration network analysis is shown in Figure 6. Author collaboration network analysis classified authors into >10 clusters, from which we identified several major research groups, mainly including Humbert *et* *al*., Galie *et* *al*., Kiely *et* *al*., and Jing *et* *al*.

**Table 4.** Top 10 prolific authors in the area of pulmonary arterial hypertension from 2011 to 2020.

Rank	Author	Number of Publications	Country
1	Marc Humbert	150	France
2	Gerald Simonneau	84	France
3	Olivier Sitbon	62	France
4	David Montani	61	France
5	Ekkehard Gruenig	46	Germany
6	Christophe Guignabert	44	France
7	Werner Seeger	43	Germany
8	Nicholas W Morrell	43	England
9	Marius M Hoeper	42	Germany
10	Anna R Hemnes	40	US

**Table 5.** Top 10 prolific authors in the area of pulmonary arterial hypertension in analyzing 100 most cited papers from 2011 to 2020.

Rank	Author	Number of Publications	Country
1	Gerald Simonneau	14	France
2	Marc Humbert	11	France
3	Adam Torbicki	8	Poland
4	Anton Vonk Noordegraaf	8	Netherlands
5	Christopher P Denton	8	England
6	Nazzareno Galie	8	Italy
7	Michael D Mcgoon	7	USA
8	Ekkehard Gruenig	7	Germany
9	Oliver Distler	5	Switzerland
10	Dinesh Khanna	5	USA

### 3.4 Contribution of journals

The top 10 journals, according to the number of citations, are listed in Table 6. The *Circulation* was the most cited journal, followed by the *Journal of the American College of Cardiology* and the *American Journal of Respiratory and Critical Care Medicine*, with 3,895, 3,406, and 3,170 citations, respectively. Furthermore, the top 10 journals on the list each had more than 1,000 citations, namely, the *Circulation* (3,895), the *Journal of the American College of Cardiology* (JACC) (3,406), the *American Journal of Respiratory and Critical Care Medicine* (3,170), the *European Respiratory Journal* (3,044), the *Chest* (3,023), the *New England Journal of Medicine* (2,564), the *European Heart Journal* (2,265), the *Circulation Research* (1,771), the *Plos One* (1,580) and the *Journal of Clinical Investigation* (1,436). The 2020 impact factor of these journals ranged from 3.24 to 91.245, among which almost all had an impact factor exceeding 10, except for *Chest* and *Plos One*. By the JCR partition analysis, Q1 was 90 and Q3 was 10% in this ranking. Except for *Plos One*, the other journals in the top 10 were included in the ‘Cardiovascular’ and ‘Respiratory categories’.

**Table 6.** Top 10 cited journals from 2011 to 2020.

Rank	Journal	Total Citation	Impact Factor	Journal Citation Reports
1	Circulation	3,895	29.69	Q1
2	Journal of the American College of Cardiology	3,406	24.094	Q1
3	American Journal of Respiratory and Critical Care Medicine	3,170	21.405	Q1
4	European Respiratory Journal	3,044	16.671	Q1
5	Chest	3,023	9.41	Q1
6	New England Journal of Medicine	2,564	91.245	Q1
7	European Heart Journal	2,265	29.983	Q1
8	Circulation Research	1,771	17.367	Q1
9	Plos One	1,580	3.24	Q3
10	Journal of Clinical Investigation	1,436	14.808	Q1

### 3.5 Analysis of keywords

**Figure 7.**
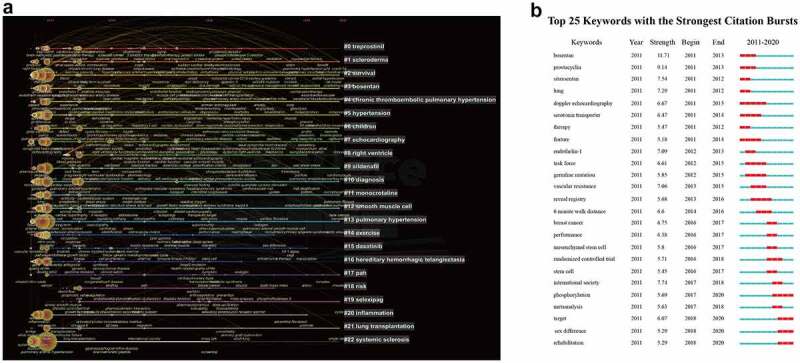
Timeline view and keywords burst. (a) Timeline view of keywords related to pulmonary arterial hypertension. (b) Keywords burst.

The keywords can reflect the hotspots and frontiers in a particular field. A total of 689 keywords were identified, which were grouped into 23 large clusters: ‘treprostinil’, ‘scleroderma’, ‘survival’, ‘bosentan’, ‘chronic thromboembolic pulmonary hypertension’, ‘hypertension’, ‘children’, ‘echocardiography’, ‘right ventricle’, ‘sildenafil’, ‘diagnosis’, ‘monocrotaline’, ‘smooth muscle cell’, ‘pulmonary hypertension’, ‘exercise’, ‘dasatinib’, ‘hereditary hemorrhagic telangiectasia’, ‘PAH’, ‘risk’, ‘selexipag’, ‘inflammation’, ‘lung transplantation’, and ‘systemic sclerosis’. The timeline of clustering indicated that ‘scleroderma’, ‘survival’, ‘bosentan’, ‘chronic thromboembolic pulmonary hypertension’, ‘hypertension’, ‘children’, ‘echocardiography’, ‘sildenafil’, ‘diagnosis’, ‘monocrotaline’, ‘smooth muscle cell’, and ‘pulmonary hypertension’ were the most important areas of PAH research, whereas ‘selexipag’ was an emerging hotspot in PAH studies (Figure 7(a)). Burstness detection allows the recognization of keywords of particular interest to the relevant scientific community at a given time. Figure 7(b) showed the map of the top 25 keywords with the strongest citation burst, in which the blue line represents the time interval and the red line represents the duration of citation burst, indicating the change of research hotspots over time. Keywords worth noting in the first five years (2011–2015) included ‘bosentan’, ‘prostacyclin’, ‘sitaxsentan’, ‘lung’, ‘Doppler echocardiography’, ‘serotonin transporter’, ‘therapy’, ‘feature’, ‘endothelin-1’, ‘task force’, ‘germline mutation’, ‘vascular resistance’, ‘reveal registry’, and ‘6-minute walk distance’. Recently (2016–2020), ‘breast cancer’, ‘performance’, ‘mesenchymal stem cell’, ‘randomized controlled trial’, ‘stem cell’, ‘international society’, ‘phosphorylation’, ‘meta-analysis’, ‘target’, ‘sex difference’, and ‘rehabilitation’ were the main research hotspots. ‘Bosentan’, together with ‘prostacyclin’, ranked first and second with a strength of 11.71 and 9.14 respectively, were regarded as hotspots from 2011 to 2013. In addition, ‘phosphorylation’, ‘target’, ‘sex difference’, and ‘rehabilitation’ have attracted lots of attention in the past three years.

## 4. Discussion

Bibliometric analysis is an excellent method that provides experienced researchers with systematic and visual knowledge structures and helps new scientific researchers to get the general trends in their field of study [19]. Herein, we perform the bibliometric knowledge analysis on the field of PAH during the last decade.

During the studied time period, the current analysis of PAH research shows a general increasing trend in PAH publications, and the number of articles was nearly 4 times more than that of reviews in the document type. It reflects that the researchers focused more on original studies rather than reviews in the area of PAH. Despite these achievements over the years, little is known about the specific mechanism of the development of PAH. The current drugs for PAH only improve symptoms and quality of life but not long-term outcomes [20], that is to say, there is no specific drug for the disease. To better treat PAH, scientists are willing to spend more effort investigating the underlying mechanisms of the pathogenesis of PAH and finding effective therapeutic targets. As a result, original studies account for approximately 80% of PAH-related publications last decade.

TC is an important index that can reflect the influence of an article and the degree of attention to its inherent scientific problems to a certain extent. Of the 10 most influential studies, eight were clinically relevant and the most cited of them were clinical guidelines on PAH. There are two possible explanations for the overfocus on clinical problems: first, PAH remains a devastating disease, and second, basic research on PAH may have reached a bottleneck period. Hence, there is an urgent need for a group of researchers who can take the discipline forward again.

The number of papers published in a certain research field is considered to be a significant index to appraise the scientific research level of a country or institution [21]. As for the country, the United States contributed the most publications, followed by China, Japan, and European countries or regions such as England, Germany, and France. This could be due to the powerful scientific research ability of the United States and Europe, as well as the large population base of China. In analyzing the top 100 cited studies, however, there was a big shake-up at the top of the rankings, with the exception of the United States, which remains No. 1. France and Germany held the second and third places respectively. It is most likely that the incidence and prevalence of PAH were high in France and Germany [22]. China ranked second in the articles number but ninth in TCs, suggesting the quality of studies published by China was not satisfactory. This was likely due to the lack of scientific research conditions in China in the past few years. However, in recent years, as China’s national strength has grown and China has invested heavily in scientific research, its research platform has gradually improved, resulting in an increasing number of high-quality articles produced in China [23]. Based on these, there is every reason to believe that China’s scientific strength and the quality of its articles will one day attract the attention of the world. As for institutes, Hôpital Bicêtre had been the most published institution around the world, followed by Vanderbilt University, and the Chinese Academy of Medical Sciences. Marc Humbert, Gerald Simonneau, and Olivier Sitbon led the world in the number of publications and citations in the field of PAH. They can be considered to be leaders in the field of PAH research. Intriguingly, when we delved into the relationship between these three people, we were pleasantly surprised to find that they all came from the same institution.

The worth of transnational cooperation in promoting innovation and resolving new and unmet challenges is widely recognized around the world [24]. As mentioned above, a lot of countries in the world conducted cooperative research in the field of PAH research from 2011 to 2020. Our study manifested that the United States was the country with the most frequent international cooperation, highlighting its influence on PAH. Meanwhile, China was second only to the United States in international cooperation, indicating that China’s influence in PAH could not be underestimated and it actively carried out international cooperation in the field of PAH. Hôpital Bicêtre carried out extensive cooperation with various leading scientific research institutions worldwide in PAH research, but interestingly, the Chinese Academy of Medical Sciences, which also ranked among the top three in the number of papers published, was mainly engaged in in-depth cooperation with Chinese institutions. The Chinese Academy of Medical Sciences, as one of the most famous research institutions in China, should cooperate with influential research institutions from all over the world, but this is not the case. The reason for this contradiction may be that cooperation between China and other countries and regions was relatively dispersed rather than concentrated. It was a good sign because only in this way could China’s scientific strength blossom. The partnership among different countries, institutions, and authors is a key driver for developing the most reliable large-scale trials [13].

The journal indices generated from the bibliometric analysis can give researchers a dependable reference to search the literature and submit manuscripts [25,26]. Our analysis showed that *Circulation* was the most cited journal. *Circulation* is one of the top publications in the cardiovascular field, known for the depth and breadth of its expertise in cardiology. It paid more attention to basic research in the area of PAH. Generally speaking, basic scientific research was the cornerstone and motivity to promote the development of scientific research in a specific field. Thus, it was unsurprising that *Circulation* had become the benchmark for PAH research. JACC was the second most cited journal, and the articles on PAH published by JACC were mainly clinical research and public health research during the past decades. Hence, JACC was complementary to *Circulation* in literature type, and JACC, together with *Circulation*, led the progress of PAH research in this era.

Keywords can reflect immediate information about the topic in certain research [27]. Considering the heterogeneity of PAH studies, we divided keywords into 23 clusters *via* co-occurrence analysis. It is challenging to develop a discussion of all clusters, so we will only discuss clusters related to the drug therapy of PAH. Cluster 0 was labeled as ‘treprostinil’. Treprostinil, a chemically stable prostacyclin (PGI_2_) analogue [28], was approved by the Food and Drug Administration (FDA) in 2002 as a treatment for PAH. It targets the PGI_2_ pathway, which plays a vital role in the pathophysiological mechanism of PAH [20]. PGI_2_ is mainly produced and secreted by vascular endothelial cells. It can bind to the corresponding receptors on various perivascular cells in physiological dose, thus maintaining the perivascular homeostasis [29]. PGI_2_ synthesis is decreased in the pulmonary artery in patients with PAH [30]. To a certain extent, administration of treprostinil to PAH patients is effective. A previous randomized controlled trial (RCT) published in 2002 has proved that it was a safe and effective treatment for PAH [31]. Another RCT, published in the *Journal of Heart and Lung Transplantation* in 2012, came to similar conclusions [32]. Cluster 3 was labeled as ‘bosentan’. Endothelin-1 (ET-1) is a potent endogenous vasoconstrictor that performs pathological or physiological functions by binding to two distinct receptors, Endothelin-1 receptor A (ET_A_) and Endothelin-1 receptor B (ET_B_). ET-1 could stimulate smooth muscle cell hyperplasia by binding to its receptor *in* *vivo* [33,34], which was regarded as a crucial link in the development of PAH. Researchers observed elevated plasma ET-1 in both animal models and patients with PAH [22,35]. Bosentan is an endothelial receptor antagonist blocking both ET_A_ and ET_B_, in which way it can improve the clinical symptoms and exercise capacity reserve of patients with PAH [36]. Cluster 9 was labeled as ‘sildenafil’. Sildenafil is a selective phosphodiesterase 5 inhibitor, previously known as UK-92,480, of which the citrate salt form is known by the trade name Viagra. It was initially used to treat angina but was later withdrawn from the treatment stage because of its various adverse reactions and relatively short half-life. Sildenafil was a potential drug for erectile dysfunction (ED) based on the multiple-dose sildenafil Phase I study [37]. Since then, there has been increasing evidence that sildenafil was effective in treating nearly all kinds of patients with ED [38–41]. Sildenafil was also an effective oral administration drug for PAH and it was proven to significantly improve the 6-min walking distance of PAH patients [42]. Cluster 19 was labeled as ‘selexipag’. Selexipag is a novel orally available drug, originally known as NS-304, which is one of the currently used drugs for PAH [43]. Selexipag and its hydrolyzate (ACT-333679), a more active form, is a prostaglandin I_2_ receptor agonist. Though comparable to prostacyclin, they are chemically different from prostacyclin [20]. Preclinical studies indicated that selexipag alleviated right ventricular remodeling, improved pulmonary circulation, and significantly increased the survival rate of rats treated with monocrotaline [44]. Olivier Sitbon *et al.* demonstrated that patients treated with selexipag had a significantly lower risk of the primary composite end point of death or PAH-related complication than those treated with placebo [45]. Though much progress has been achieved in treating PAH, the current treatment of PAH is mainly vasodilators, which mainly act on the healthy pulmonary vascular system [20]. As a result, patients can only regain exercise or functional ability without a satisfactory long-term prognosis, so there is an unmet demand for drugs that can effectively target the pathophysiological mechanisms leading to disease progression and improve the natural outcome. Recently, a growing body of evidence suggests that monotherapy is often inadequate to relieve patients’ clinical symptoms and reduce the risk of clinical exacerbation [46,47]. Early active management with combination therapy may benefit patients [47–49]. Taken together, we need to conduct further studies to discover novel drugs targeting the pathophysiological mechanisms of PAH and to explore combination therapies for PAH.

To the best of our knowledge, this is the first study to illustrate trends in PAH research over the past decade using bibliometric analysis. Moreover, our study provides extensive and in-depth directions for researchers. However, there are some limitations. First, we just analyzed the development of PAH research in the past decades, which did not represent the origin and development of the entire field of PAH research. Second, the WoSCC database is constantly updated. Something obtained from our study is essentially temporary. Third, we only included English publications in this study. It is possible that our results may differ from the actual publishing characteristics.

## 5. Conclusion

In conclusion, the annual quantity of publications on PAH showed an overall increase in the last decade. The United States was the most prolific country, while Hôpital Bicêtre also made important research achievements, which was instrumental in driving the further development of PAH. Moreover, Marc Humbert led the PAH field by publishing the most articles. Given the complex nature of cardiovascular medicine, there was a close transnational relation among countries, institutions, and authors, which was expected to foster innovation and resolve novel and unresolved challenges in the future. The results of our study bring us new ideas for the PAH-related studies and may benefit further researches on the etiology, diagnosis, and treatment of the disease.
